# Draft Genome Sequences of *Salmonella enterica* Serovar Typhimurium LT2 with Deleted Chitinases That Are Emerging Virulence Factors

**DOI:** 10.1128/genomeA.00659-17

**Published:** 2017-08-03

**Authors:** Narine Arabyan, Bihua C. Huang, Bart C. Weimer

**Affiliations:** a Department of Population Health and Reproduction, School of Veterinary Medicine, University of California, Davis, California, USA; b 100K Pathogen Genome Project, University of California, Davis, California, USA

## Abstract

Chitinases are glycosyl hydrolases that catalyze the hydrolysis of the β-1,4 linkages in complex carbohydrates and those that contain GlcNAc. These enzymes are considered emerging virulence factors during infection because the host glycan changes. This is the release of four single chitinase deletion mutants in *Salmonella enterica* serovar Typhimurium LT2.

## GENOME ANNOUNCEMENT

Chitinases are glycosyl hydrolases (GHs) that belong to the GH18 and GH19 families (1–6). GH enzymes play a significant role in virulence by altering the host glycan structure during infection and gaining access to the host epithelial cells, which results in the microbe binding to terminal monosaccharides to initiate glycan degradation on the host epithelial cell (7, 8). Chitinases are emerging virulence factors because they recognize host GlcNAc-containing glycans in mucin and other *N*-glycosylated proteins in the host membrane, which enable host association as well as glycan digestion, to gain access to the cell membrane to initiate invasion (9, 10). Glycans with GlcNAc molecules with a β-1,4-glycosidic bond (11) are found on intestinal epithelial cells (IECs) and are hydrolyzed during association (1, 10). This provides *Salmonella* spp. a method to degrade the glycan and digest the glycocalyx to establish intracellular infections.

Deletion of chitinase genes in *Listeria monocytogenes* led to a reduction in bacterial counts in the liver and spleen of infected mice (12). An adherent-invasive *Escherichia coli* (AIEC) LF82 deletion of the *chiA* gene significantly reduced the adhesion to IECs compared to that of the wild type (13). Furthermore, AIEC LF82 interacted with an *N*-glycosylated chitin-binding protein (CHI3L1) on the host cell to mediate close interaction between the host membrane and bacterial cell, which is regulated in animal models of colitis and in human inflammatory bowel diseases (IBDs) (14). Microarray analysis showed that *SL0018* (*chiA*) gene in the *Salmonella* SL1344 strain was strongly induced during the infection of murine macrophage cells (15, 16). These data indicate that chitinases relandscape the host glycan to promote the attachment of bacteria to the host cells through the interaction with mucin or *N*-glycosylated glycans during association. The genus *Salmonella* contains four chitinases that were derived from bacteria and phages. Park et al. (8) also demonstrated that *Salmonella* initiates glycan relandscaping during infection via host gene expression changes and microbe grazing to degrade the glycan, making these enzymes important for infection.

The 100K Pathogen Genome Project (http://www.100kgenomes.org) is a large-scale sequencing consortium that offers the use of new next-generation sequencing methods to provide cutting-edge methods for pathogen detection and control in the food supply. This project is focused on sequencing genomes of bacteria from the environment, plants, animals, and humans worldwide, providing new insights into the genetic diversity of pathogens and the microbiome. Four chitinase deletions (*ΔSTM0018, ΔSTM0233*, *ΔSTM0907*, and *ΔSTM1869A*) were constructed in the Weimer laboratory (University of California, Davis) (7) as described by Datsenko and Wanner (17). Cultures were prepared for sequencing as described previously (18–25). Genome sequences were *de novo* assembled using CLC Workbench version 6.5.1 with default parameters (18).

### Accession number(s).

All sequences are publicly available and can be found at the 100K Pathogen Genome Project BioProject (NCBI PRJNA186441) in the Sequence Read Archive (http://www.ncbi.nlm.nih.gov/sra). NCBI GenBank accession numbers for the genome assemblies are listed in Table 1.

**TABLE 1:** *Salmonella enterica* serovar Typhimurium LT2 chitinase deletion mutants

GenBank accession no.	SRA accession no.	Isolate name	Gene deleted	Enzyme activity	No. of contigs	Coverage (×)	Total genome size (bp)	No. of coding sequences
MZNQ00000000	SRR5288763	BCW_8404	*ΔSTM0018*	Exochitinase	61	139	4,893,048	4,810
MXBA00000000	SRR5288762	BCW_8406	*ΔSTM0233*	Endochitinase	63	162	4,894,557	4,808
MXBB00000000	SRR5288761	BCW_8409	*ΔSTM0907*	Prophage chitinase	61	188	4,895,634	4,808
MZYL00000000	SRR5288732	BCW_8417	*ΔSTM1869A*	Putative chitinase	63	177	4,895,461	4,811
